# Comparative evaluation of acute respiratory distress syndrome in patients with and without H1N1 infection at a tertiary care referral center

**DOI:** 10.4103/0019-5049.76602

**Published:** 2011

**Authors:** Tanvir Samra, Mridula Pawar, Amlendu Yadav

**Affiliations:** Department of Anesthesia and Intensive Care, Dr. Ram Manohar Lohia Hospital, Connaught Place, New Delhi, India

**Keywords:** ARDS, critical care, H1N1, influenza, pandemic

## Abstract

H1N1 subtype of influenza A virus has clinical presentation ranging from mild flu like illness to severe lung injury and acute respiratory distress syndrome (ARDS). The aim of our study was to compare the demographic characteristics, clinical presentation, and mortality of critically ill patients with (H1N1+) and without H1N1 infection (H1N1-). We retrospectively analyzed medical charts of patients admitted in “Swine Flu ICU” with ARDS from August 2009 to May 2010. Real-time reverse transcriptase polymerase chain reaction (RT-PCR) assay was used for detection of H1N1 virus in the respiratory specimens. Clinical data from 106 (H1N1, 45; H1N1+, 61) patients was collected and compared. Mean delay in presentation to our hospital was 5.7 ± 3.1 days and co-morbidities were present in two-fifth of the total admissions. Sequential Organ Failure Assessment (SOFA) score of patients with and without H1N1 infection was comparable; 7.8 ± 3.5 and 6.6 ± 3.1 on day 1 and 7.2 ± 4.5 and 6.5 ± 3.1 on day 3, respectively. H1N1+ patients were relatively younger in age (34.2 ± 12.9 years vs. 42.8 ± 18.1, *P* = 0.005) but presented with significantly lower PaO_2_:FiO_2_ ratio (87.3 ± 48.7 vs. 114 ± 51.7) in comparison to those who subsequently tested as H1N1. The total leucocyte counts were significantly lower in H1N1+ patients during the first four days of illness but incidence of renal failure (*P* = 0.02) was higher in H1N1+ patients. The mortality in both the groups was high (H1N1+, 77%; H1N1, 68%) but comparable. There was a mean delay of 5.7 ± 3.1 days in initiation of antivirals. Patients with H1N1 infection were relatively younger in age and with a significantly higher incidence of refractory hypoxia and acute renal failure. Mortality from ARDS reported in our study in both the groups was high but comparable.

## INTRODUCTION

Acute respiratory distress syndrome (ARDS) is characterized by acute hypoxemic respiratory failure and bilateral pulmonary infiltrates following a systemic or pulmonary insult (extrapulmonary vs. pulmonary ARDS).[1] The emergence of influenza A (H1N1) virus as a pandemic has introduced a new etiological risk factor for pulmonary ARDS.[2] The aim of this study was to compare the clinical course and outcome of patients with and without H1N1 infection admitted in intensive care unit with ARDS.

## METHODS

Data was collected after analyzing medical case files, treatment charts, radiologic, and laboratory reports of patients with ARDS[3] admitted in “Swine flu ICU” from August 2009 to May 2010. Information regarding age, sex, clinical features, indices of oxygenation (PaO_2_/FiO_2_ ratios) and ventilation, laboratory investigations, mortality, and parameters needed to calculate Sequential Organ Failure Assessment (SOFA)[4] score were noted. H1N1 influenza virus was detected using real-time reverse transcriptase polymerase chain reaction (RT-PCR)[5] in respiratory specimens collected at the time of admission in ICU. Patients with positive bacterial and fungal cultures in endotracheal aspirates or active tuberculosis or those with a duration of stay of less than 24 hours were excluded from the study.

Only conventional modes of mechanical ventilation were available in our ICU. High frequency oscillatory ventilation (HFOV), extracorporeal membrane oxygenation (ECMO), prone positioning, and recruitment manoeuvers were not used. Ventilatory management initiated on admission to ICU was in accordance with guidelines mentioned in the ARDSnet trial.[6] Propensity of H1N1 virus to invade lung tissue and cause severe lung injury led to fluctuating clinical and haemodynamic parameters, which necessitated the attending physician to make multiple changes in the ventilator settings. Therapeutic dose of oseltamivir and broad spectrum antibiotics were administered empirically upon admission and subsequent changes were based on reports of culture/sensitivity. Supportive care included chest and limb physiotherapy, nebulisation with bronchodilators and mucolytics, and enteral feeding by nasogastric tube. Infusions of midazolam and fentanyl were titrated to maintain deep sedation. Muscle relaxants were administered to facilitate mechanical ventilation in patients with high pulmonary inflation pressures. Patients with septic shock were managed according to guidelines published in surviving sepsis campaign.[7]

Haematological and biochemical investigations, arterial blood gas analysis (ABG), and chest X-ray were done at the time of admission, daily, and as and when required. Blood, tracheal, and urine cultures were sent on admission, twice a week, and during febrile spikes. Electrocardiography, pulse oximetry, non-invasive and invasive blood pressure, central venous pressure, and urine output were monitored.

Infection control measures were followed and N-95 and other personal protective equipments (PPE) were used by health care workers (HCW).

### Statistical analysis

The statistical sofware, SPSS 13 (SPSS Inc., Chicago, IL) was used for data analysis. Discrete variables were expressed as counts (percentage) and continuous variables as means ± SD or median. For demographic and clinical characteristics of patient, difference between two groups was assessed using chi-square test or Fischer’s exact test for categorical variables and Student’s t test or Mann–Whitney U-test for continuos variables. *P* ≤ 0.05 was considered statistically significant.

## RESULTS

One hundred and seventy-two patients were admitted in the ICU with diagnosis of ARDS; 106 (H1N1, 45; H1N1 ^+^, 61) were included for further analysis out of which 6 were pregnant ladies. Sixty-six patients were excluded in view of ambiguity in diagnosis and etiology of lung injury, and the presence of insufficient information. Out of 45 patients tested negative for H1N1, 15 were positive for influenza A (non-H1N1 subtype).

Patients with H1N1 infection were administered 150 mg BD of oseltamivir on admission, which was continued for 10 days or till survival in ICU. Reductions in dose were done for patients with renal failure. Empirical antibiotics administered during course of illness included beta-lactam, fluoroquinolones, and macrolides. None of the patients had prior immunisation with seasonal influenza vaccine.

Invasive mechanical ventilation using low tidal volume lung protective ventilation was used in all patients on arrival to ICU. However, the attending physician had to make multiple changes in each patient to ensure haemodynamic and clinical stability. Pressure control-synchronous intermittent mandatory ventilation (PC-SIMV) was the most frequently used weaning mode after initial stabilization. Positive end expiratory pressure (PEEP) of 10–15 cm of H_2_O and 15–20 cm H_2_O was used in 65% and 35% of ventilated patients, respectively.

Being a tertiary care hospital, majority of admissions in our ICU (85% of H1N1+ and 83% of H1N1) were of patients referred from one or more peripheral health centres. Percentage of patients received intubated was higher in H1N1+ group (26% vs. 12%). There was a significant delay in transfer of H1N1+ patients from ward to ICU (1.2 ± 1.4 days in H1N1+ vs. 0.6 ± 0.6 days in H1N1, *P* = 0.01).

Demographic and clinical characteristics were comparable except age, PaO_2_:FiO _2_ ratio, incidence of acute renal failure, and total leucocyte counts [Table 1]. Refractory hypoxia, haemodynamic compromise, acidosis, acute renal failure (ARF), multi organ dysfunction syndrome (MODS), and disseminated intravascular coagulation (DIC) were the main complicating factor in descending order of frequency developing in H1N1+ patients during stay in ICU. H1N1 patients had similar complications but in the following descending order; haemodynamic compromise, refractory hypoxia, acidosis, DIC, MODS, and ARF. Continuous renal replacement therapy was used in two H1N1+ patients. Secondary bacterial infection with *Klebsiella pneumonia* and acinetobacter was detected in two and empyema thoracis with *Candida albicans* developed in one H1N1 patient.

**Table 1:** Demographic and clinical characteristics of patients admitted in ICU

Variable	H1N1- (n = 45)	H1N1+ (n = 61)	*P* value
Age (years)	42.8 ± 18.1	34.2 ± 12.9	0.005^*^
Gender, Male	24 (53)	31 (50.8)	0.80
GCS on admission	10.4 ± 4.6	10.7 ± 4.7	0.78
Duration of illness (days)	5.6 ± 3.2	5.7 ± 3.1	0.82
SOFA score			
SOFA (Day 1)	6.6 ± 3.1	7.8 ± 3.5	0.08
SOFA (Day 3)	6.5 ± 3.1	7.2 ± 4.5	0.43
PaO_2_:FiO_2_	114 ± 51.7	87.3 ± 48.7	0.008^*^
PaO_2_ at time of intubation (mm Hg)	49.6 ± 11.4	45.1 ± 14.0	0.08
Hypotension at time of admission	8 (17.8)	8 (13.1)	0.51
Adverse events			
1. Refractory hypoxia	12 (26)	27 (44.2)	0.04^*^
2. Acidosis (pH < 7.1)	9 (20)	18 (29.5)	0.27
3. Inotropic requirement during ICU stay			
Single inotrope	6 (13.3)	11 (18.0)	0.20
Double inotrope	11 (24.4)	13 (21.3)	0.70
4. ARF	4 (8.8)	16 (26.2)	0.02^*^
5. MODS	5 (11.1)	12 (19.6)	0.24
6. DIC	5 (11.1	3 (0.05)	0.23
Laboratory investigations			
Total leucocyte count (TLC)			
TLC -Day 1 (cells/mm^3^)	22,450 ± 7,648	9,750 ± 6,122	0.001^*^
TLC -Day 2 (cells/mm^3^)	18,150 ± 7,868	12,050 ± 6,666	0.008^*^
TLC -Day 3 (cells/mm^3^)	24,400 ± 6,002	10,650 ± 4,067	0.001^*^
TLC -Day 4 (cells/mm^3^)	20,000 ± 7,980	12,000 ± 7,7737	0.048^*^
Anaemia	5 (11.1)	12 (19.7)	0.24
Thrombocytopenia	6 (13.3)	9 (14.8)	0.84

Values are mean ± SD or n (%) *P* ≤ 0.05 was considered statistically significant. GCS: Glasgow Coma Scale; ICU: Intensive care unit; SOFA: Sequential organ failure assessment; DIC: Disseminated intravascular coagulation; ARF: Acute renal failure; MODS: Multiple organ dysfunction syndrome; TLC: Total leucocyte count

Elevated lactate dehydrogenase (LDH) and aminotransferases levels were seen in only 3 patients with diagnosis of swine flu and myocarditis. Deranged liver function tests were seen in very few patients; three in the H1N1+ group in comparison to only one in the H1N1 group.

Bilateral patchy alveolar opacities (predominantly basal) affecting three or all four quadrants was the most common radiographic feature in H1N1+ patients admitted in the ICU.

**Figure 1:**
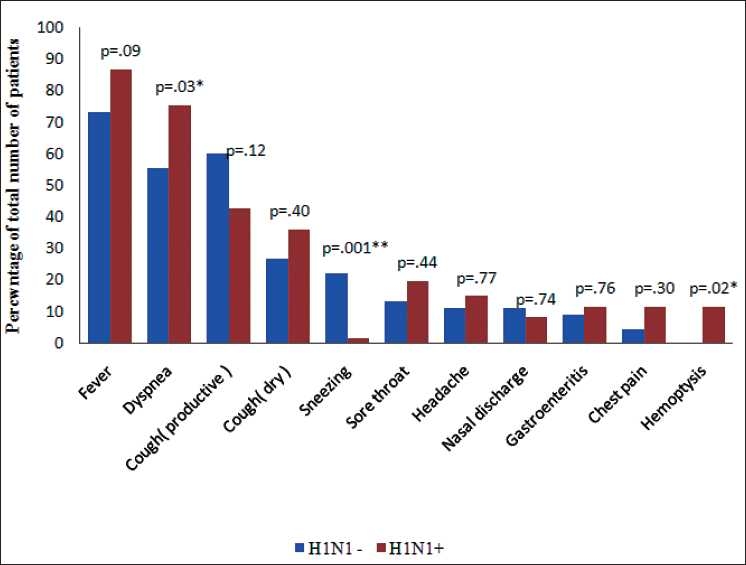
Clinical features on admission. *P*0 ≤ 0.05 was considered statistically significant, *P* ≤ 0.05 was considered statistically significant

Clinical features of both the groups are shown in Figure 1. Majority presented as febrile flu like illness but a small percentage were admitted with atypical features like gastroenteritis.

**Figure 2:**
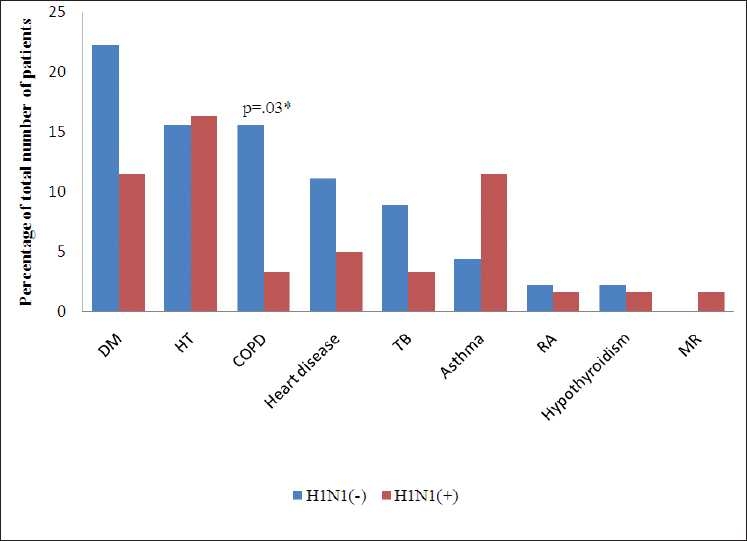
Co-morbid conditions, DM, diabetes mellitus; HT, hypertension; COPD, chronic obstructive pulmonary disease; TB, tuberculosis; RA, rheumatoid arthritis; MR, mental retardation, Heart disease in H1N1(-): coronary artery disease (4), coronary artery disease and cerebrovascular accident (1). Heart disease in H1N1(+): atrial septal defect (1), rheumatic heart disease (1), coronary artery disease (1). *P*.≤ 0.05 was considered statistically significant

Percentage of patients with co-morbidities was similar in both the groups (42% in H1N1, 47% in H1N1+; *P* = 0.44, Figure 2).

**Figure 3:**
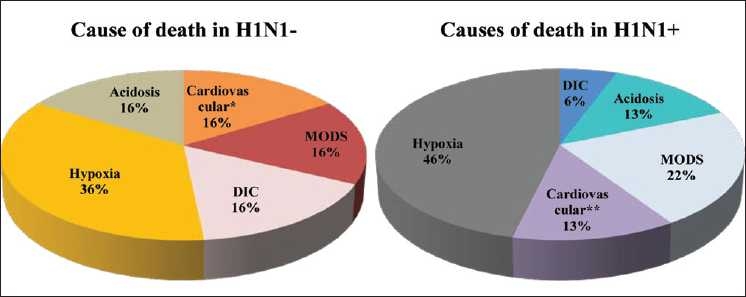
Cause of death in H1N1+ and H1N1 patients, ^*^3 patients had been resuscitated from cardiac arrest in ward; 1 had myocardial ischaemia; 1 was in congestive cardiac failure at time of admission. ^*^^*^1 patient had been resuscitated from cardiac arrest in ward; 3 had myocarditis; 3 were in congestive cardiac failure at time of admission

Mortality was 77% in the H1N1+ group and 69% in the H1N1 group (*P* = 0.35) with refractory hypoxia as the most common cause of death Figure 3.

## DISCUSSION

Our report compares the clinical features and outcome of ARDS patients with and without H1N1 infection admitted in “Swine Flu ICU” in our Hospital. A lot of published work from other countries sharing their experience of 2009 flu pandemic exist but reports from developing countries like India with high population densities and limited health care facilities are scarce.[8]

One striking feature in our study has been the high mortality in both H1N1+ and H1N1 patients. Previous published work from multidisciplinary ICU in a developing country like Nepal[9] has reported a mortality of 73.9% for patients with systemic inflammatory response syndrome and mean SOFA score of >7. In view of this, we had also anticipated a high mortality as mean SOFA score of H1N1+ and H1N1 patients was 7.8 ± 3.5 and 6.6 ± 3.1 on day 1 of ICU admission and 7.2 ± 4.5 and 6.5 ± 3.1 on day 3, respectively.

Case fatality rates of 30%, 21%, 17%, and 41% have been reported in critically ill patients admitted with H1N1 infection in Michigan,[10] Australia, New Zealand,[11] Canada[12] and Mexico,[13] respectively. It is to be noted that these studies report combined mortality of patients with and without ARDS and acute lung injury, whereas in our study we have included only those with ARDS. Study from a better equipped ICU in Bangalore[8] reports a survival of 84% at end of 28 days and is the first to highlight the clinical features and mortality of critically ill patients with H1N1 infection in India. But favourable factors present in their study population were better lung compliance, single organ failure, low mean APACHE II score, and use of prone position and High Frequency Oscillation for cases with refractory hypoxia.

Pooled mortality for patients with both pulmonary and extrapulmonary ARDS due to various precipitating factors is 44% for observational studies, which is again lower than what we have reported.[14] Factors like multiple referrals, delay in transfer to ICU and initiation of antiviral medicine and a conservative approach to intubation and mechanical ventilation could be contributing factors for high mortality in our study. Another differentiating feature between our study and previously published reports[10–13] is the lack of prior seasonal influenza vaccination and non-availability of HFOV, ECMO, zanamivir, and recombinant activated protein C in our ICU.

Limitations of our study were in its observational design and inability to evaluate the process of care prior to admission in ICU. Inability to isolate the primary pathogen for pneumonia and ARDS in 30 H1N1 patients was another limitation. Tests for detection of other viruses like rhinovirus, respiratory syncytial virus, adenovirus, parainfluenza, etc. could not be performed due to lack of resources and no organism could be isolated from repeated bacterial or fungal cultures of respiratory, urine, and blood specimens. All patients had empirically received antibiotics prior to sample collection, and thus, validity of culture reports is also doubtful.

## CONCLUSION

Commensurate with world wide experience, most of our H1N1+ patients were young adults. Dyspnea, haemoptysis, lower total leucocyte counts, refractory hypoxia, and acute renal failure was present in significantly greater percentage of patients with swine flu. Mortality due to ARDS secondary to viral pneumonia was higher (77% in H1N1+; 69% in H1N1) than the reported pooled mortality due to all causes of ARDS (44%). There is a need to conduct studies focussing on specific subsets of patients with ARDS rather than the broad categories of pulmonary and extrapulmonary lung injury.
